# Open reduction and internal fixation offers lower hip‐related complications compared to stem revision in Vancouver B2 fractures around cemented polished tapered femoral stems

**DOI:** 10.1002/jeo2.70179

**Published:** 2025-02-13

**Authors:** Olof Sköldenberg, Sebastian Mukka, Michael Axenhus, Carl‐Johan Hedbeck, Martin Magnéli

**Affiliations:** ^1^ Unit of Orthopaedics, Department of Clinical Sciences Danderyd Hospital Karolinska Institutet Danderyd Sweden; ^2^ Department of Surgical and Perioperative Sciences (Orthopaedics) Umeå University Umeå Sweden

**Keywords:** arthroplasty, fractures, ORIF, periprosthetic, revision

## Abstract

**Purpose:**

Periprosthetic femoral fractures (PFFs) after total hip arthroplasty (THA) are increasing, particularly Vancouver B2 fractures around cemented polished tapered femoral stems. Open reduction and internal fixation (ORIF) are more frequently used in comparison to the traditional stem revision to deal with these complex fractures. This observational study aims to compare the outcomes of ORIF versus stem revision in the treatment of Vancouver B2.

**Methods:**

A retrospective cohort study was conducted at Danderyd Hospital, Stockholm, from 2008 to 2022, including 157 patients (mean age 83.4 ± 7.0 years, 59% females) with a surgically treated Vancouver B2 fractures with an intact bone‐cement interface. The study assessed the immediate and long‐term outcomes of ORIF versus stem revision, examining post‐operative complications, reoperation rates, and implant survivorship.

**Results:**

Among the 157 patients, 37 were treated with ORIF and 120 with stem revision. The ORIF group, which consisted of older patients and had a higher prevalence of cognitive dysfunction, experienced no hip‐related adverse events. In contrast, the revision group had a 17.8% incidence of adverse events. Mortality within 90 days was significantly higher in the ORIF group (24%) compared to the revision group (4%) (*p* = 0.0007). One‐year mortality was also higher in the ORIF group (32%) than in the revision group (15%) (*p* = 0.03).

**Conclusions:**

ORIF presents as a viable option for managing Vancouver B2 fractures in the proximity of a polished tapered stem when anatomical reduction is possible. The less invasive surgery provides potential advantages in patient outcomes and resource utilization. Further research is warranted to aid in the development of treatment guidelines.

**Level of Evidence:**

III

**Abbreviations**
ASA: American Society of Anesthesiologists
BMI: body mass index
CT: computed tomography
FNF: femoral neck fracture
LCP: locking compression plate
MP: modular prosthesis
NCB: non‐contact bridging
ORIF: open reduction and internal fixation
PFF: periprosthetic femoral fractures
PJI: periprosthetic joint infection
REDCap: Research Electronic Data Capture
SD: standard deviation
STROBE: Strengthening the Reporting of Observational Studies in Epidemiology
THA: total hip arthroplasty

## INTRODUCTION

Periprosthetic femoral fractures (PFFs) following total hip arthroplasty (THA) are becoming increasingly prevalent in orthopaedic practice, largely due to the rising numbers of primary and revision hip arthroplasties being performed globally [13]. The Vancouver classification system offers a structured approach to categorizing these fractures based on anatomical location, prosthetic stability, and bone stock quality, aiding in the decision‐making process for treatment [17]. Vancouver B2 fractures, which occur around a loose femoral stem, present a particular challenge because they involve intrinsic prosthetic instability, typically requiring more complex interventions beyond simple fracture fixation [2].

The two main strategies for managing Vancouver B2 fractures are open reduction and internal fixation (ORIF) or stem revision. While ORIF focuses on addressing the fracture alone, the inherent instability associated with the loose stem in B2 fractures often necessitates stem revision to restore biomechanical integrity and ensure long‐term success [6].

Cemented polished tapered femoral stems, such as the Exeter (Stryker) or CPT (Zimmer‐Biomet), have been associated with a higher incidence of postoperative B2 fractures compared to anatomical stems, potentially because the tapered design acts as a wedge, contributing to femoral splitting [3, 14]. Emerging research has explored management strategies for B2 fractures around these stems, with evidence suggesting that ORIF may be a viable alternative to revision in cases where the bone‐cement interface remains intact, and the fracture can be anatomically reduced [16, 20]. ORIF is associated with reduced blood transfusion requirements and lower risks of revision surgery compared to stem revision, offering potential advantages in both clinical outcomes and healthcare resource utilization.

Recent studies, including those by Stoffel et al. and Di Martino et al., have highlighted the benefits of ORIF for Vancouver B2 fractures [4, 23]. This study aims to evaluate the impact of ORIF on patient outcomes in comparison to stem revision in the management of Vancouver B2 fractures.

## PATIENTS AND METHODS

### Design and setting

This retrospective cohort study was performed between 2008 and 2022 at the Orthopedic Department of Danderyd Hospital in Stockholm, Sweden. Danderyd Hospital is a university hospital affiliated with the Karolinska Institute and provides medical care to a catchment area of approximately 800,000 inhabitants. Study data were managed using REDCap electronic data capture tools [9] and collected up until September of 2022 with a minimum of 1 year follow‐up after surgery. Ethical approval was obtained for the study. The STROBE guidelines for reporting of observational cohorts were followed [5].

### Participants

Patients were identified through the local surgical planning system, medical records and the Swedish Arthroplasty register. A consecutive series of all surgically treated periprosthetic Vancouver B2 fractures were included. Cases had an intact bone‐cement interface around a fractured, loose cemented, collarless, polished tapered stem. We excluded periprosthetic fractures around other types of stems (cemented and uncemented), patients with known (before fracture) loosening of the stem, atypical and pathological fractures.

### Surgery

At the start of surgery, the stability of all stems was tested (if not immediately obvious from pre‐operative X‐rays) through direct manipulation of the stem/cement interface and direct visual view of the hip. Fractures with stable (non‐loose) stems were classified as Vancouver B1 fractures and were not eligible for inclusion in the study. All surgeons were experienced general consultant orthopaedic surgeons with either a focus on traumatology or hip arthroplasty surgery. Ultimately, the decision to revise or fix the implant in situ were up to individual surgeons’ choice.

#### Revision group

For stem revision procedures, two primary approaches were employed. One utilized an uncemented, distally anchored modular femoral stem (MP, Link Sweden). The other involved a choice between the cemented femoral stem options: the CPT (Zimmer‐Biomet), the Lubinus SP II (Waldemar Link), or the cemented, distally anchored modular femoral stem (MP, Waldemar Link).

**Figure 1:**
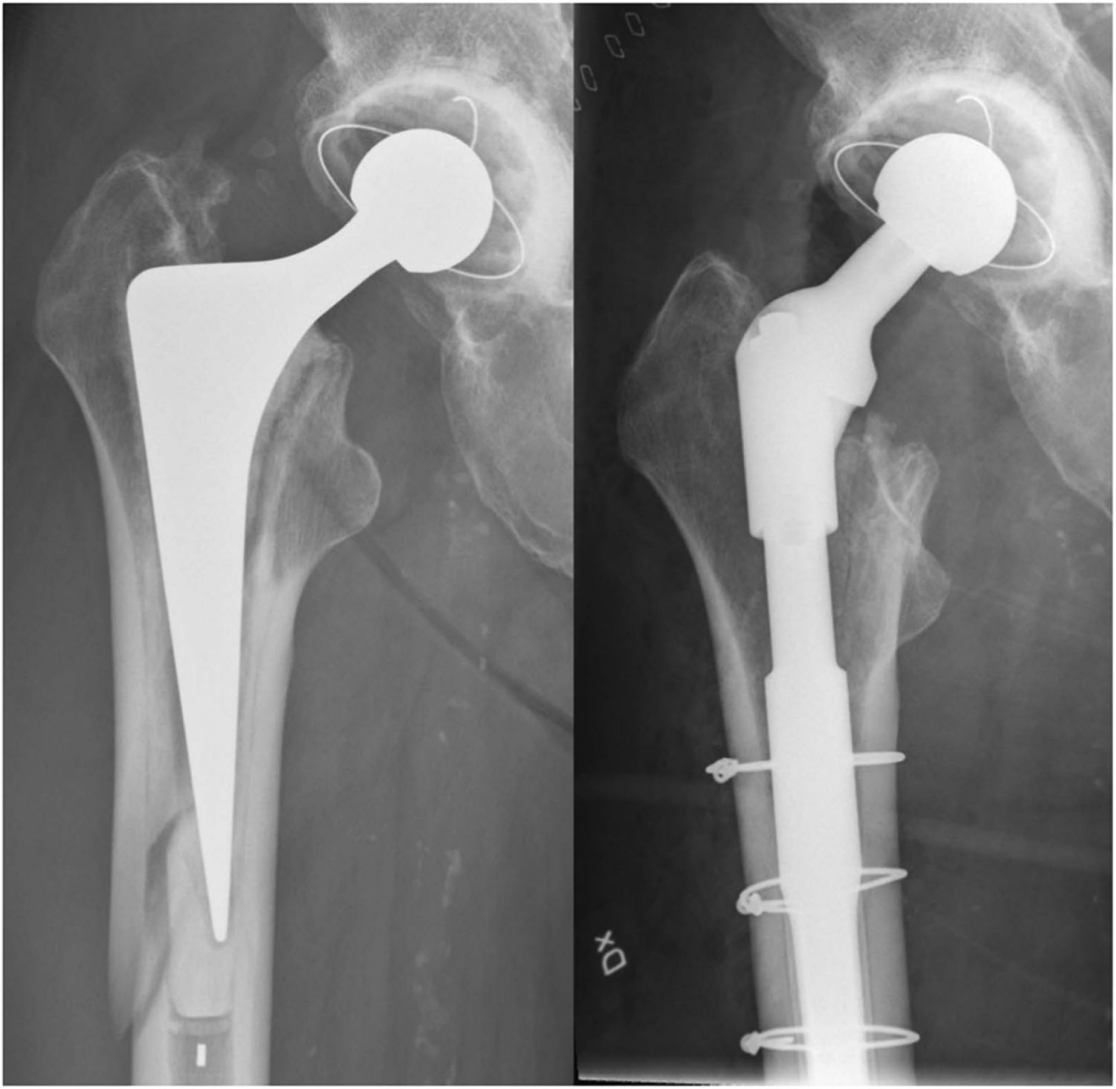
Vancouver B2 reverse clamshell fracture treated with a distally anchored stem revision.

In terms of the cemented stems, they were categorized based on length. Stems shorter than 150 mm were classified as ‘short’, while those exceeding 150 mm were labelled ‘long’. With the uncemented stems, meticulous care was exercised to ensure complete removal of the existing bone cement from the femoral canal, emphasizing the proximal segment. Conversely, for cemented stems, a ‘cement‐in‐cement’ revision technique was employed. This entailed minimal cement removal, just enough to accommodate the new stem within the femoral canal (Figure 1).

Additionally, some surgeons opted for supplementary stabilization techniques. This could involve the use of cerclage wires and proximal femoral plates, depending on the clinical assessment. Postoperatively full weight‐bearing without restrictions were used.

#### ORIF group

**Figure 2:**
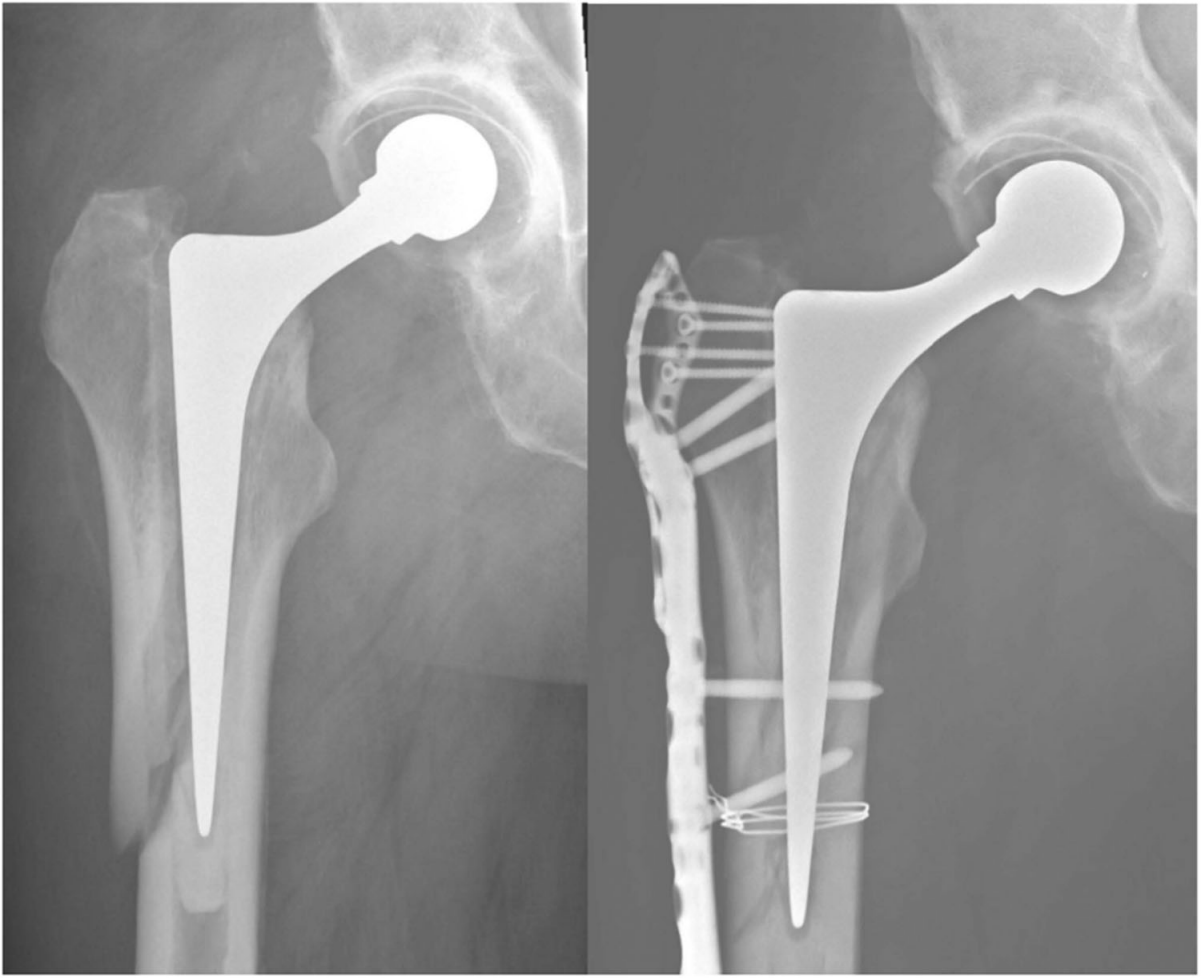
Vancouver B2 reverse clamshell fracture treated with open reduction and internal fixation.

For patients treated with ORIF, the primary aim was to achieve as close to an anatomical reduction of the fracture as possible without exposing (i.e., opening) the joint. A fracture was deemed appropriate for ORIF if there was no symptomatic loosening at the bone‐cement interface, no significant comminution, or no evidence of stem subsidence into its centralizer. In instances where the primary surgeon was not specialized in hip revision surgeries, a dedicated revision hip surgeon was available on call to potentially intervene if necessary. A lateral approach exposed the fracture site, followed by an inspection of the bone‐cement interface. When a satisfactory anatomical reduction was achieved, fixation was accomplished using plate and screws, occasionally supplemented with cerclage cabling. Subsequently, a lateral plate was applied, either the NCB from Zimmer‐Biomet or the LCP proximal femoral plate from Synthes Depuy (Figure 2).

Strut allografts were not used in any of the procedures. Weight‐bearing was individualized depending on fracture type and was ultimately up to the surgeon, most patients were allowed partial weight‐bearing for up to 6 weeks after surgery and then full weight bearing as tolerated.

### Outcomes and data collection

The main outcome was hip‐related complications, reoperations and clinical outcome. Periprosthetic joint infection (PJI) was defined as local signs of infection that involve the joint prosthesis and surrounding tissues. We used the Swedish personal identification number to gather data over the study duration via a combination of our hospital's surgical and medical database and routine follow‐up appointments. A digital case report form was used to gather all study data. Additionally, to detect any reoperations conducted externally, we used the Swedish Hip Arthroplasty Register to identify any reoperations outside the Stockholm regional area.

### Variables

For the study we collected data including age, sex, cognitive impairment, American Society of Anesthesiologists (ASA) score, primary indication for surgery, type of arthroplasty (THA or hemiarthroplasty), time to radiological healing of fracture, and all hip complications leading to reoperation including open surgery with revision of implants as well as closed reduction of dislocations. A fracture was considered healed if the radiologist reported healing in the radiology report. The periprosthetic fractures were classified according to the proposed new classification system for Vancouver B2 fractures [10]. All fractures were classified by the first author on plain radiographs into either burst, clamshell, reverse clamshell or spiral fractures by the first author. In 42 cases CT was used in combination with plain x‐ray to aid in the fracture classification. The clinical and radiographic outcomes for the patients with PPF were evaluated by a combination of a medical chart review and follow‐up visit. The outcome was graded as: ‘good’ in patients with a radiographically healed fracture and no or little walking impairment, ‘intermediate’ in patients with a healed fracture but impaired walking ability, and ‘poor’ in patients with an unhealed fracture and/or a severely impaired walking ability.

### Sample size

No separate power calculation was performed prior to this descriptive study.

### Statistical analyses

All statistical analyses were conducted using R (version 4.3.1). Demographic data were summarized as percentages for categorical variables and as means, medians, and standard deviations for continuous variables. Differences in patient demographics were assessed using the *χ* ^2^ test for categorical variables and the *t*‐test for continuous variables. The differences in 90‐day and 1‐year mortality between the ORIF and revision groups were evaluated using Fisher's exact test.

To account for the competing risk of death when assessing the risk of reoperation, we employed the Fine and Gray sub distribution hazard model. This model estimates the sub distribution hazard ratio (SHR), reflecting the probability of reoperation over time while considering death as a competing event. Covariates in the model included age, ASA score, body mass index (BMI), cognitive dysfunction, and treatment type (ORIF vs. revision). Results were reported as hazard ratios with corresponding 95% confidence intervals and *p* values.

All tables were created using the Table 1 package. Survival probability curves were generated with the survival and Survminer packages, while competing risk calculations were performed using the cmprsk package.

**Table 1:** Descriptive characteristic of included patients.

	ORIF (*N* = 37)	Revision (*N* = 120)	*p* value
Gender			
Male	14 (37.8%)	51 (42.5%)	0.755
Female	23 (62.2%)	69 (57.5%)	
Mean (SD)	85.2 (7.97)	82.9 (6.55)	0.11
Median [min, max]	86.0 [70.0, 104]	83.0 [66.0, 97.0]	
ASA classification			
ASA 2	6 (16.2%)	32 (26.7%)	0.43
ASA 3	29 (78.4%)	82 (68.3%)	
ASA 4	2 (5.4%)	6 (5.0%)	
BMI			
Mean (SD)	24.1 (4.03)	24.7 (4.45)	0.403
Median [min, max]	23.0 [16.0, 32.0]	24.0 [15.0, 39.0]	
Cognitive dysfunction			
No	24 (64.9%)	98 (81.7%)	0.097
Possible/uncertain	2 (5.4%)	4 (3.3%)	
Definitive	11 (29.7%)	18 (15.0%)	
Primary indication for surgery			
Osteoarthritis	27 (73.0%)	67 (55.8%)	0.15
FNF	9 (24.3%)	43 (35.8%)	
Aseptic loosening of THA	1 (2.7%)	10 (8.3%)	

*Note*: *p* Values derived from *χ* ^2^ test for categorical variables and *t*‐test for continuous variables.

Abbreviations: ASA, American Society of Anesthesiologists; BMI, body mass index; FNF, femoral neck fracture; ORIF, open reduction and internal fixation; THA, total hip arthroplasty.

## RESULTS

**Figure 3:**
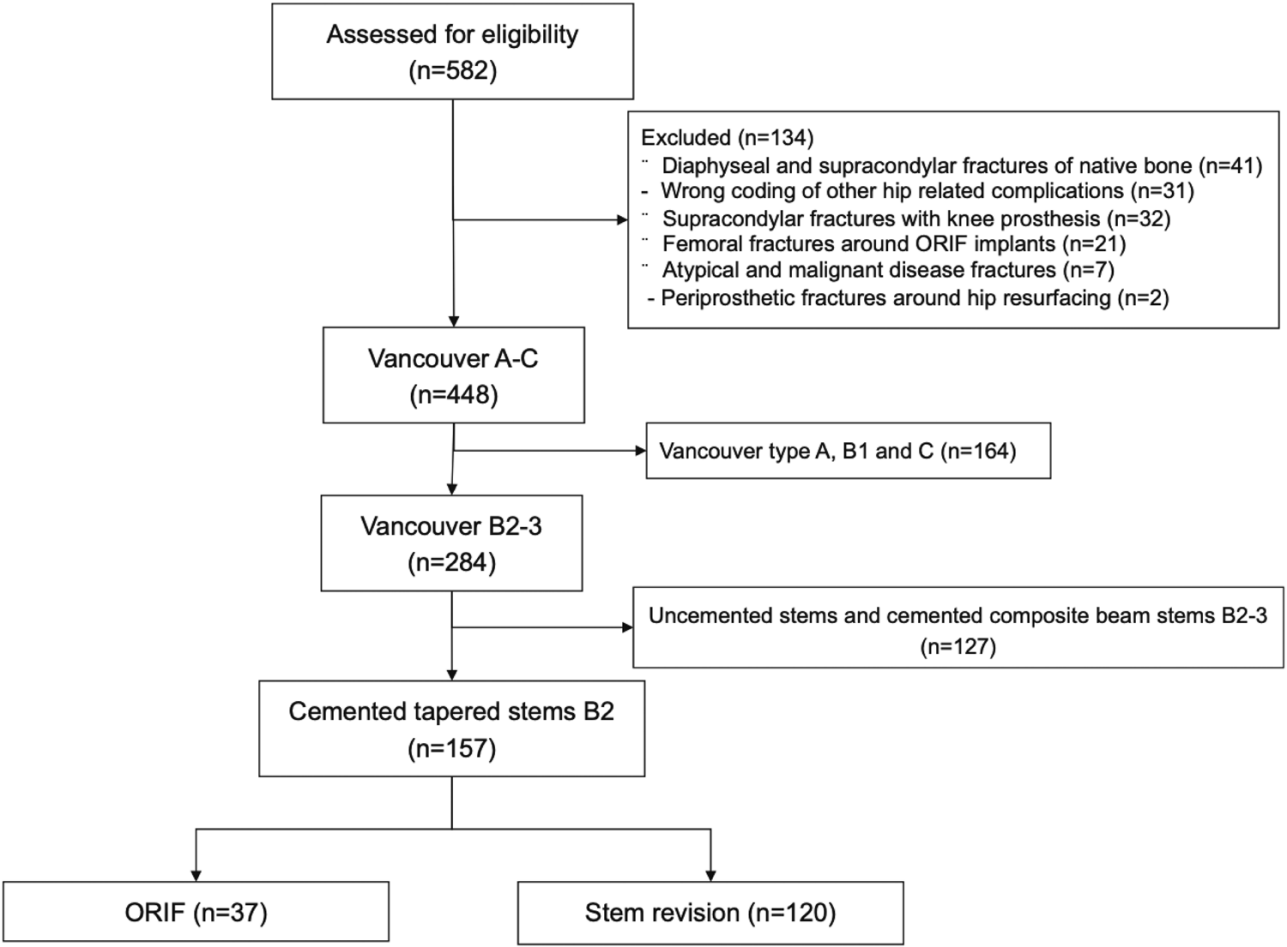
Flow of patients in the study.

A total of 157 patients (mean age 83.4 ± 7.0 years, 59% females) were included in the study. Of these, 37 patients underwent ORIF, while 120 were treated with stem revision. Baseline characteristics of the two groups were largely similar, with no statistically significant differences in terms of sex, age, ASA class, BMI, cognitive dysfunction status, or surgical indications. However, the ORIF group was slightly older, with 10% more patients in ASA 3–4 categories and a higher proportion of cognitive dysfunction, indicating a likely preference for ORIF in frailer elderly patients (Figure 3, Table 1).

### PFF characteristics and treatment

The most common fracture pattern was spiral fractures (39%). In the revision group, uncemented long modular stems were most frequently used (35%) (Table 2).

**Table 2:** Fracture types, treatment, clinical outcomes and adverse events.

	ORIF (*N* = 37)	Revision (*N* = 120)	*p* value
Type of fracture			
Burst fracture (axe split fracture)	3 (8.1%)	21 (17.5%)	0.121
Clamshell (medial)	6 (16.2%)	34 (28.3%)	
Reverse clamshell (lateral)	10 (27.0%)	19 (15.8%)	
Spiral	18 (48.6%)	43 (35.8%)	
Not possible to categorize	0 (0%)	3 (2.5%)	
Stem subsidence	23 (62.2%)	70 (58.3%)	0.824
Fracture dislocation	18 (48.6%)	86 (71.7%)	0.014
Treatment, implant			
Modular long uncemented stem	0 (0%)	44 (36.7%)	
Modular long cemented stem	0 (0%)	3 (2.5%)	
Normal uncemented stem	0 (0%)	0 (0%)	
Standard cemented stem	0 (0%)	37 (30.8%)	
Long cemented stem	0 (0%)	36 (30.0%)	
Plate fixation only	33 (89.2%)	0 (0%)	
Cerclage wiring only	4 (10.8%)	0 (0%)	
Clinical outcome			
Good outcome (healed without apparent sequelae)	20 (54.1%)	75 (62.5%)	0.001
Intermediate outcome (healed with impairment to walking ability)	7 (18.9%)	34 (28.3%)	
Poor outcome (major limitation/bedridden/confined to wheelchair)	0 (0%)	7 (5.8%)	
Deceased as a direct consequence of periprosthetic fracture	7 (18.9%)	3 (2.5%)	
Missing	3 (8.1%)	1 (0.8%)	
Adverse events			
Dislocation	0	17 (14.2%)	0.034
PJI	0 (0%)	9 (7.5%)	0.19
Non union	0 (0%)	1 (0.8%)	1
New PFF (Vancouver C)	0 (0%)	2 (1.7%)	1
Subsidence or stem loosening	0 (0%)	7 (5.8%)	0.295
Non‐union of greater trochanter	0 (0%)	3 (2.5%)	0.776

Abbreviations: ORIF, open reduction and internal fixation; PFF, periprosthetic femoral fracture; PJI, periprosthetic joint infection.

### Treatment results and adverse events

A total of 29 patients (18%) experienced adverse events (AEs), all of whom were in the revision group. Some patients had a combination of AEs, such as PJI combined with dislocation (*n* = 4) or PJI combined with stem subsidence (*n* = 3). The most frequent AE was dislocation (*n* = 17), followed by PJI (*n* = 9). Seven patients experienced excessive stem subsidence, three of which led to dislocation. Other AEs included nonunion of the greater trochanter (*n* = 3), new distal periprosthetic fractures (*n* = 2), and nonunion in the diaphysis (*n* = 1).

Out of the 27 AEs that required surgical treatment, 24 involved open surgeries: cup revision or articulation change (*n* = 10), stem revision (*n* = 9), debridement, antibiotics, and implant retention (DAIR, *n* = 7), exchange of modular stem components (*n* = 3), and plate fixation of distal periprosthetic fractures (*n* = 2). Closed reduction of a dislocated prosthesis was performed in three cases.

### Competing risks and mortality

The Fine and Gray sub distribution hazard model showed that revision surgery was associated with a 78% higher hazard of reoperation compared to ORIF, with the result being marginally significant (HR = 1.78, 95% CI: 1.00–3.15, *p* = 0.050). ASA class (HR = 1.79, *p* = 0.004) and cognitive dysfunction (HR = 2.13, *p* < 0.001) were significant predictors of reoperation, while age and BMI were not (Table 3).

**Table 3:** Hazard model for association with reoperation.

Characteristic	HR	95% CI	*p* value
ASA class	1.65	1.04 ‐ 2.63	0.035
Age	1.02	0.97 ‐ 1.06	0.4
Cognitive dysfunction	2.26	1.75 ‐ 2.93	<0.001
BMI	0.97	0.91 ‐ 1.03	0.3
Treatment			
ORIF	efef		
Revision	1.57	0.87 ‐ 2.84	0.13

Abbreviations: ASA, American Society of Anesthesiologists; BMI, body mass index; CI, confidence interval; HR, hazard ratio; ORIF, open reduction and internal fixation.

### Mortality rates

**Figure 4:**
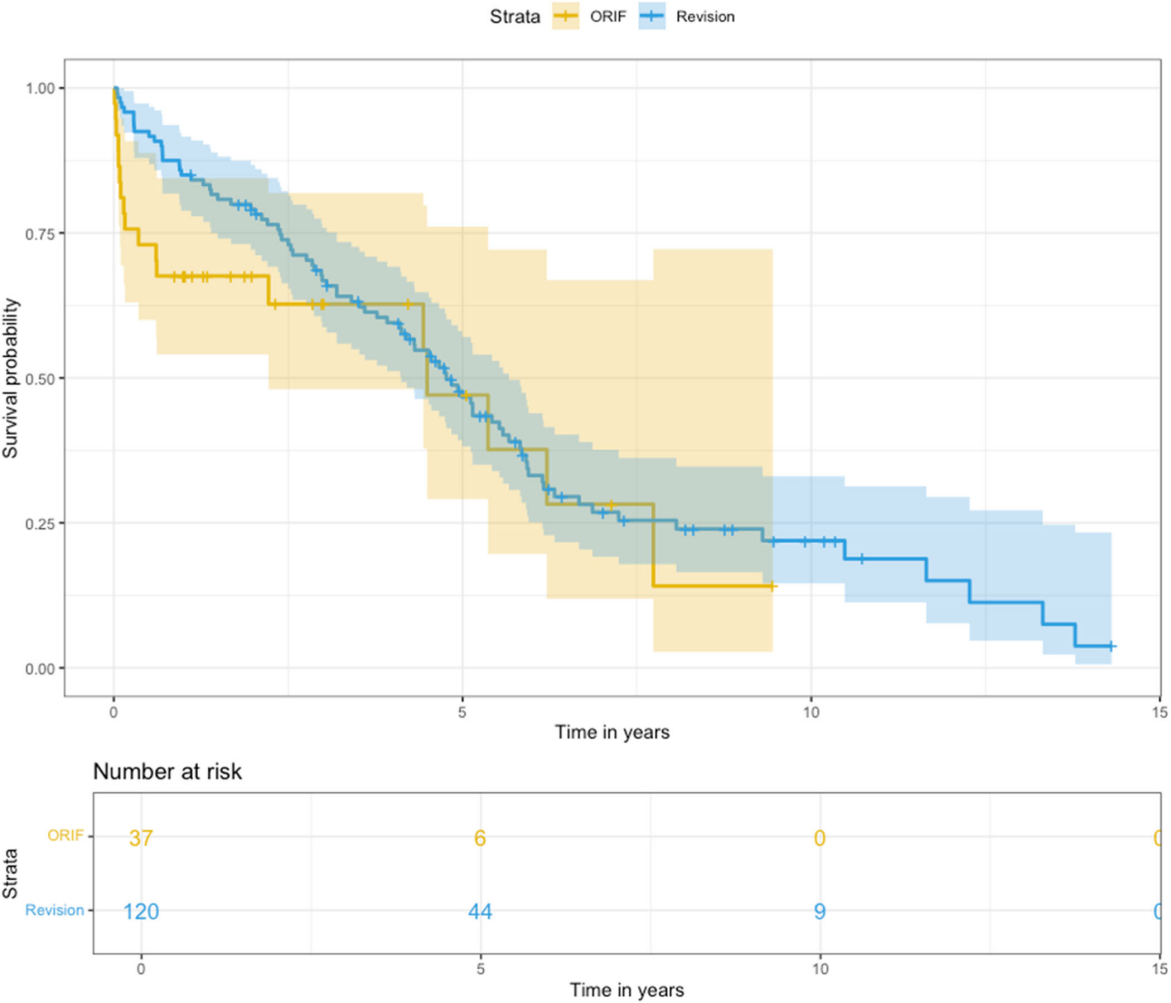
Patient survival probability of open reduction and internal fixation versus revision group up to 15 years post‐surgery.

There was a significant difference in 90‐day mortality following periprosthetic fracture surgery. Mortality was 24% in the ORIF group compared to 4% in the revision group (*p* = 0.0007). One‐year mortality was 32% in the ORIF group and 15% in the revision group (*p* = 0.03). Kaplan–Meier survival analysis further emphasized these differences, showing consistently lower survival rates in the ORIF group compared to the revision group (Figure 4).

## DISCUSSION

This retrospective cohort study demonstrates that ORIF is a viable option for treating Vancouver B2 fractures around polished tapered femoral stems, provided anatomical reduction is achievable. The less invasive nature of ORIF offers potential benefits, including lower surgical morbidity, fewer hip‐related complications, and more efficient utilization of healthcare resources compared to stem revision. Due to the higher mortality observed in the ORIF group, comprising predominantly older and frailer patients, ORIF should be regarded as a favourable option for managing Vancouver B2 fractures, particularly in terms of hip‐related outcomes and resource utilization. This approach is especially suitable for patients where minimizing surgical invasiveness is a priority, provided anatomical reduction is achievable.

In this study, ORIF was associated with no reoperations or hip‐related adverse events, while the revision group exhibited a 17.8% incidence of complications, including dislocations and periprosthetic joint infections. Additionally, ORIF was performed on older, frailer patients with higher ASA scores and cognitive dysfunction, yet still achieved favourable outcomes. Mortality within 90 days and one year was significantly higher in the ORIF group, likely reflecting the frailty of this patient population.

Overall, these findings suggest that ORIF, when feasible, can provide effective management for this fracture type, particularly in patients where reducing surgical invasiveness is a priority. Further research is needed to refine patient selection criteria and develop evidence‐based treatment guidelines for Vancouver B2 fractures in this specific setting.

The Vancouver classification is a reliable and valid system for the classification of PFFs and guides surgeons in the treatment of these complex fractures [17]. However, the Vancouver classification can be difficult to use for fractures in the proximity of cemented polished tapered stems. Taper slip stems are loose within the original cement mantle and as most Vancouver B fractures in these stems involve a cement mantle fracture, these stems are loose and thus graded as a B2 fracture. However, the entire cement mantle has been proposed to be considered a part of the implant and if the bone‐cement interface is intact, the stem is not considered loose [12]. Maggs et al. has proposed an amendment to the classifications system to include a subclassification of B2 fractures around cemented femoral stems to include B2W, where the cement‐bone interface is intact, and B2L, where the cement is loose [12].

ORIF of Vancouver B2 fractures in the proximity of polished tapered stems has been reported previously [8, 9, 11, 16, 20, 21, 22]. 23/09/2023 06:08:00Patients considered for ORIF are low‐demand older patient (>65 years) with a well‐cemented polished tapered stem with an intact cement‐bone interface, without signs of previous loosening and at least some partial stem‐cement attachment, suffering a non‐comminute fracture possible to achieve an anatomical reduction of the fracture [20]. The achievement of an anatomical reduction of the integrated cement mantle restores the principle of the polished taper slip and an adequate clinical outcome without stem revision [19]. A failure to achieve anatomic reduction predisposes to stem subsidence and loosening [8].

There are some specific fracture patterns that are identified which are either impossible to anatomically reduce and other that are reducible but associated with fixation failure or early stem loosening [9]. The proposed specific patterns are comminute metaphyseal split fractures, fractures of the calcar that are difficult to address, fractures with loosening at the bone‐cement interface, the stem has been significantly impacted distally into the centralizer which, in certain cases, is difficult to disimpact the stem and reduce the fracture proximally [18]. A recent, large observational study comparing ORIF and stem revision in Vancouver B fractures in the proximity of polished tapered stems found that stem revision was associated with a longer waiting time to surgery, more blood transfusion, and a higher hip complication rate. These findings are on par with the conclusion of a recent systematic review and meta‐analysis comparing ORIF and revision arthroplasty for Vancouver B2 fractures [7]. In the present study, dislocation was the most commonly occurring complication which is in concordance with other reports in the literature [1, 9, 15].

The limitations of the present study include the retrospective study design, the skewed number of patients in each treatment group wither fewer patients treated with ORIF, the differences in baseline characteristics are a limitation of the present study. However, this study adds data regarding possible treatment options for Vancouver B2 fractures in the proximity of polished tapered stems that are eligible for both ORIF and stem revision.

## CONCLUSION

This retrospective cohort study identified that ORIF presents as a viable option for managing Vancouver B2 fractures in the proximity of a polished tapered stem when anatomical reduction is possible. The less invasive surgery provides with potential advantages in patient outcomes and resource utilization. Further research is warranted to aid in the development of treatment guidelines.

## AUTHOR CONTRIBUTIONS

*Conceptualization and Validation*: Olof Sköldenberg, Sebastian Mukka, Martin Magnéli and Carl‐Johan Hedbeck. *Data curation, formal analysis, Investigation and Methodology*: Michael Axenhus, Sebastian Mukka, Olof Sköldenberg and Martin Magnéli. *Project administration and resources*: Michael Axenhus and Olof Sköldenberg. *Software*: Michael Axenhus, Olof Sköldenberg and Martin Magnéli. *Supervision*: Olof Sköldenberg, Carl‐Johan Hedbeck and Martin Magnéli. Visualization: Michael Axenhus and Martin Magnéli. *Writing—original draft*: Olof Sköldenberg. Writing—review and editing: Michael Axenhus, Sebastian Mukka, Martin Magnéli and Carl‐Johan Hedbeck.

## CONFLICT OF INTEREST STATEMENT

The authors declare no conflicts of interest.

## ETHICS STATEMENT

This study was approved by the ethical board at Karolinska Institutet (2009/97‐31/2).

## Data Availability

Data are available from the corresponding author on reasonable request.
